# The Effect of Anakinra in Hospitalized Patients with COVID-19: An Updated Systematic Review and Meta-Analysis

**DOI:** 10.3390/jcm10194462

**Published:** 2021-09-28

**Authors:** Konstantinos G. Kyriakoulis, Anastasios Kollias, Garyphallia Poulakou, Ioannis G. Kyriakoulis, Ioannis P. Trontzas, Andriani Charpidou, Konstantinos Syrigos

**Affiliations:** Third Department of Medicine, School of Medicine, Sotiria Hospital, National and Kapodistrian University of Athens, 11527 Athens, Greece; taskollias@gmail.com (A.K.); gpoulakou@gmail.com (G.P.); ioannis.kyriakoulis@gmail.com (I.G.K.); john-tron@hotmail.com (I.P.T.); dcharpidou@yahoo.gr (A.C.); ksyrigos@med.uoa.gr (K.S.)

**Keywords:** anakinra, COVID-19, COVID-19 therapeutics, immunomodulatory treatment, meta-analysis, mortality, updated

## Abstract

The role of immunomodulatory agents in the treatment of hospitalized patients with COVID-19 has been of increasing interest. Anakinra, an interleukin-1 inhibitor, has been shown to offer significant clinical benefits in patients with COVID-19 and hyperinflammation. An updated systematic review and meta-analysis regarding the impact of anakinra on the outcomes of hospitalized patients with COVID-19 was conducted. Studies, randomized or non-randomized with adjustment for confounders, reporting on the adjusted risk of death in patients treated with anakinra versus those not treated with anakinra were deemed eligible. A search was performed in PubMed/EMBASE databases, as well as in relevant websites, until 1 August 2021. The meta-analysis of six studies that fulfilled the inclusion criteria (*n* = 1553 patients with moderate to severe pneumonia, weighted age 64 years, men 66%, treated with anakinra 50%, intubated 3%) showed a pooled hazard ratio for death in patients treated with anakinra at 0.47 (95% confidence intervals 0.34, 0.65). A meta-regression analysis did not reveal any significant associations between the mean age, percentage of males, mean baseline C-reactive protein levels, mean time of administration since symptoms onset among the included studies and the hazard ratios for death. All studies were considered as low risk of bias. The current evidence, although derived mainly from observational studies, supports a beneficial role of anakinra in the treatment of selected patients with COVID-19.

## 1. Introduction

The course of coronavirus disease 2019 (COVID-19) is divided in two main phases: the viral and the host inflammatory response phases [1,2,3]. During the second phase, a dysregulation of the immune system might occur in a subset of patients leading to a cytokine storm and immune hyperactivation cascade [1]. In these cases, antiviral treatment has little to offer, and thus the role of immunomodulatory agents has been of increasing interest [4,5].

Anakinra is an interleukin-1 inhibitor that has been shown to offer benefits alone or in combination with other agents for the treatment of diseases characterized by a cytokine storm (e.g., pediatric secondary hemophagocytic lymphohistiocytosis, and macrophage activation syndrome) [6,7]. It plays an important role in the inhibition of the cytokine storm cascade and can offer benefits to selected patients with COVID-19 [1]. Four recently published meta-analyses indicated that anakinra administration in hospitalized patients with COVID-19 and moderate to severe disease offered significant benefits in terms of mortality and the risk of intubation [8,9,10,11]. However, these analyses included mainly unadjusted effect estimates [8,9,10,11]. Unadjusted analyses might be significantly affected by several confounding factors since treatment options in COVID-19 may differ according to patient characteristics and the severity of the disease. Interestingly, the most recent study included an individual patient-level meta-analysis in a subgroup of 895 patients, which allowed a multivariate analysis and showed a significant adjusted risk reduction with the use of anakinra [11].

The aim of the present study was to conduct an updated systematic review and meta-analysis on the impact of anakinra on the survival of hospitalized patients with COVID-19. To compensate for the nature of derived evidence, this analysis included randomized studies and observational ones presenting adjusted hazard ratios for several confounders.

## 2. Materials and Methods

### 2.1. Search Strategy

An updated systematic review and meta-analysis was performed according to the preferred reporting items for systematic reviews and meta-analyses (PRISMA) guidelines [12]. A systematic search of PubMed and EMBASE databases was performed until 1 August 2021, using the following search algorithm: (“coronavirus 2019” OR “2019-nCoV” OR “SARS-CoV-2” OR “COVID-19” OR COVID OR COVID19) AND anakinra. Articles were also identified from reference lists of previously conducted relevant systematic reviews and meta-analyses and relevant papers and websites through the snowball procedure. The study selection was performed independently by two investigators (K.G.K. and I.G.K.). Disagreements were resolved by consensus with a senior author (A.K.).

### 2.2. Study Selection

Eligible studies were full-text articles in English language including ≥15 patients (not case series) that had a randomized design or were observational but reported exclusively adjusted hazard ratios for mortality between patients treated with anakinra versus those who did not receive anakinra. More precisely, eligible studies were: (i) randomized studies, (ii) observational studies with propensity matched controls, and (iii) observational studies with multivariate analysis models (including several potential confounders such as demographics, comorbidities, laboratory indices and background treatment with other therapeutic agents).

### 2.3. Data Extraction

Two investigators (K.G.K. and I.G.K.) independently extracted and tabulated data regarding study design, the main characteristics of included populations (age, sex, number of patients treated with anakinra, number of patients that required invasive mechanical ventilation, comorbidities, symptoms duration before anakinra administration, and severity indices at baseline, such as C-reactive protein) and data regarding the outcome of interest (adjusted hazard ratio for mortality).

### 2.4. Risk of Bias Assessment

The risk of bias was assessed in terms of the selection of patients, exposure measurement, confounding factors identification, outcome measurement, methodology and analysis independently by two investigators (K.G.K. and I.G.K.). A checklist for cohort studies from the Joanna Briggs Institute Critical Appraisal Tools was used [13]. Studies fulfilling ≥8 of the quality domains were deemed as low risk of bias.

### 2.5. Certainty (Confidence) of the Outcome

The certainty of the body of evidence for the outcome of death was independently assessed by two investigators (K.G.K. and A.K.) using the grading of recommendations assessment, development and evaluation (GRADE) approach described in Chapter 14 of the Cochrane handbook for systematic reviews of interventions [14]. The certainty of evidence was deemed as high, moderate, low, or very low, depending on factors that either decrease the confidence of the outcome such as the risk of bias, the publication bias, the inconsistency, the indirectness and the imprecision of results, or factors that increase the certainty such as the large effect size, the dose response, and the effect of plausible residual confounding [15].

### 2.6. Statistical Analysis

Meta-analysis was performed using the Stata/SE 11 (Texas) software. The logarithms of adjusted hazard ratios and corresponding standard errors were used for the analysis (fixed-effects meta-analysis when I^2^ statistic value < 50%). The hazard ratio was used as the effect measure of the outcome of interest as it was reported in all included studies. Results were graphically displayed as forest plots. A meta-regression analysis was performed for assessing associations of the logarithms of the hazard ratios for mortality with the mean age, percentage of males, mean baseline C-reactive protein levels, and mean time of administration since symptoms onset. The mean values of the subgroups were combined where feasible [16]. Median (interquartile range) values were converted to mean values (standard deviation) using the appropriate formulas [17]. Heterogeneity was tested using I^2^ statistics. Publication bias was assessed by inspecting funnel plots, as well as Egger’s test (linear regression method) and Begg’s test (rank correlation method) [18,19]. Two-sided *p* values < 0.05 were considered statistically significant. Missing information was retrieved after communication with the corresponding authors.

## 3. Results

### 3.1. Literature Search and Inclusion of Studies

Four relevant meta-analyses on the impact of anakinra on the outcomes of hospitalized COVID-19 patients were identified [8,9,10,11]. Among the 28 studies included in these analyses (with significant overlap), four studies that reported adjusted hazard ratio for mortality were identified and included in our synthesis [20,21,22,23].

Regarding the updated literature search, among 1018 initially retrieved articles, one study was additionally identified to fulfill the inclusion criteria and was included in our analysis [24]. This study provided two hazard ratios for early and delayed administration of anakinra versus standard of care, respectively [24].

Finally, after a website search, the first placebo-controlled randomized trial on the effect of anakinra in hospitalized COVID-19 patients was identified, at a preprint version at the time of the search [25].

The main characteristics of the six included studies are shown in Table 1. The PRISMA 2020 checklist for the present meta-analysis is presented in the Supplementary File, Table S1. The PRISMA 2020 abstracts checklist is presented in the Supplementary File, Table S2. The PRISMA 2020 flow diagram for updated systematic reviews and meta-analyses study selection is presented in the Supplementary File, Figure S1.

### 3.2. Data Synthesis

**Figure 1:**
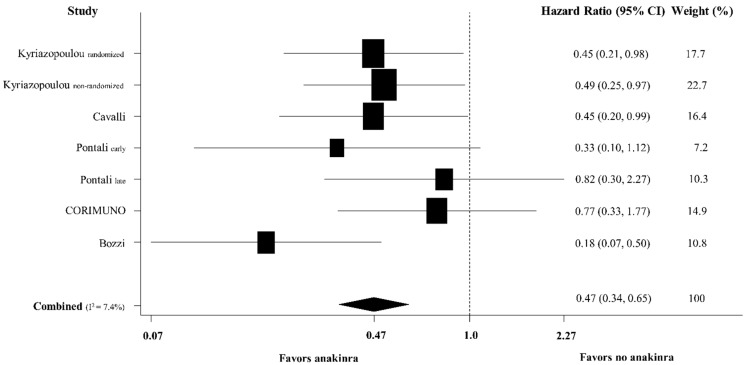
Forest plot of adjusted hazard ratios for death for patients treated with anakinra versus those who did not receive anakinra among hospitalized patients with COVID-19.

The meta-analysis of the six included studies (*n* = 1553, weighted age 64 years, male sex 66%, treated with anakinra 50%, intubated 3%) showed a pooled hazard ratio for death in patients treated with anakinra versus those who did not receive anakinra at 0.47 (95% confidence intervals [CI] 0.34, 0.65) (Figure 1). A 28-day mortality was the endpoint of interest in the majority of studies [20,21,22,25]. Most patients had moderate to severe COVID-19 (Table 1).

### 3.3. Sensitivity and Meta-Regression Analyses

Three sensitivity analyses were performed for robustness: (1) after excluding the only randomized placebo-controlled trial [25], the pooled adjusted hazard ratio remained the same at 0.47 (95% CI 0.33, 0.68); (2) after excluding the two randomized studies (a placebo-controlled and one standard of care-controlled study) [21,25], the pooled adjusted hazard ratio was similar at 0.42 (95% CI 0.28, 0.63); (3) after excluding a study that seemed to differ significantly from the others both in terms of percentage of intubated patients (33%) and in terms of the hazard ratio for mortality (0.18) [20], the pooled adjusted hazard ratio was 0.53 (95% CI 0.37, 0.74). A multivariate meta-regression analysis did not reveal any significant associations between the mean age (regression coefficient [RC] 0.17, 95% CI −0.19, 0.53), percentage of males (RC −0.05, 95% CI −0.33, 0.23), mean baseline C-reactive protein levels of the patients receiving anakinra (RC 0.001, 95% CI −0.04, 0.04), and mean time of administration since symptoms onset (RC 0.01, 95% CI −0.40, 0.42) among the included studies and the hazard ratios for death (all *p* > 0.10). In addition, there was no association between the daily dose of anakinra during the first three days of administration and the hazard ratios (RC −0.001, 95% CI −0.005, 0.002, *p* = 0.45) (the variable of the daily dose was not included in the multivariate meta-regression analysis due to insufficient observations).

### 3.4. Risk of Bias, Publication Bias and Certainty of the Evidence Assessment

All studies were deemed as having a low risk of bias. The assessment of the risk of bias of the included studies is presented in the Supplementary File, Table S3.

Egger’s test and Begg’s funnel plots did not reveal any small study effect (*p* > 0.10 for both) (Supplementary File, Figure S2).

The certainty of the evidence on the outcome of death was high and in favor of a beneficial effect of anakinra administration in hospitalized patients with COVID-19 (Supplementary File, Table S4).

## 4. Discussion

This updated meta-analysis showed about a 50% decrease in the adjusted risk of death in hospitalized patients with moderate-to-severe COVID-19 treated with anakinra compared with patients that did not receive anakinra.

Four meta-analyses have been previously conducted investigating the impact of anakinra treatment on the outcomes of hospitalized patients with COVID-19 [8,9,10,11]. These studies confirmed the safety profile of anakinra and further demonstrated a beneficial impact of this treatment in patients with mainly moderate to severe COVID-19 pneumonia along with increased inflammatory indices [8,9,10,11]. However, these meta-analyses included mainly observational studies and used unadjusted ratios for calculating pooled estimates [8,9,10,11]. A major methodological limitation inevitably accompanying observational studies is the fact that their results are influenced by the lack of randomization and the subsequent indication bias for each arm of treatment. Specifically, it seems that earlier or more aggressive and combination treatment or higher doses have been selectively administered to patients with critical COVID-19. However, the effectiveness of such interventions might be muffled by the adverse outcome in cases with irreversible establishment of severe complications [26]. In addition, the selection of candidate patients and the optimal time of each intervention might also play a major role in preventing adverse events [27]. The meta-analysis by Kyriazopoulou et al. had the advantage of individual data meta-analysis (and thus of adjusted analyses) in a subsample and confirmed the findings of the unadjusted analyses [11].

In our updated meta-analysis only high-quality studies providing adjusted ratios were included. Most studies were non-randomized observational studies designed to compare the standard of care treatment plus anakinra versus the standard of care treatment alone [20,22,23,24]. One study was randomized but not placebo controlled [21] and only one study was a placebo controlled double-blind trial [25]. Interestingly, in one observational study both early and late anakinra administration were investigated [24]. In the early administration group, anakinra was administered after a mean of 9 days of symptoms initiation, while in the late administration group anakinra was administered after 15 days of symptoms initiation. Early administration tended to have a greater beneficial effect compared with late administration; however, both hazard ratios were not at the level of statistical significance (0.33 (95% CI 0.10, 1.12) and 0.82 (95% CI 0.30, 2.27), respectively). Although the sample size was limited and robust conclusions cannot be drawn, it appears that the proper time of anakinra administration might play an important role.

Another important point regarding COVID-19 therapeutics is the proper patient selection for each therapeutic regimen. Selection criteria in most studies included increased inflammation indices and/or severe COVID-19. Indeed, the baseline characteristics of the included studies indicated that most patients needed any type of oxygen supply, and their admission CRP levels were increased. Thus, in most cases a moderate to severe pneumonia accompanied by a hyperinflammation syndrome had already been established. Anakinra, an interleukin-1 inhibitor, plays an important role in the inhibition of the cytokine storm cascade and can apparently offer benefits to this group of patients. Interestingly, in the studies by the research group of Giamarellos-Bourboulis [23,25] a biomarker indicating a high probability of future hyperinflammation syndrome (soluble urokinase plasminogen activator receptor (suPAR)) was used to guide therapeutic decisions, possibly allowing the administration of anakinra earlier in the course of COVID-19 before clinical establishment of severe disease. In the meta-analysis by Kyriazopoulou et al. subgroup sensitivity analyses were performed and showed that anakinra was more effective in mortality reduction in patients with CRP higher than 100 mg/L [11]. In our meta-regression analysis, there was no association between baseline CRP levels in patients receiving anakinra and hazard ratios, but it should be highlighted that in general, a meta-regression analysis examines the associations between the outcome and several characteristics which are aggregate and summarized at the level of the study which in turn introduces ecological bias. A tailored and individualized approach to indicate (i) the optimal time of administration and (ii) the group of patients that will benefit the most, appears to be of paramount importance.

One of the main limitations of the current analysis is the paucity of randomized controlled trials on the role of anakinra on the outcomes of patients with COVID-19. However, the inclusion of studies that provided adjusted hazard ratios might at least partially compensate for this limitation. Furthermore, the findings were consistent in several sensitivity analyses.

## 5. Conclusions

Anakinra seems to have a beneficial role as a therapeutic agent for selected patients with COVID-19, especially those with moderate or severe pneumonia accompanied by increased levels of inflammatory indices. Findings of previous observational studies and meta-analyses of unadjusted ratios were confirmed by the current analysis of adjusted hazard ratios derived from high quality (low risk of bias) studies. Additional placebo-controlled randomized trials are needed to further evaluate the efficacy of this intervention.

## Figures and Tables

**Table 1:** Main characteristics and findings of included studies.

Study	Design	*n*	Treated with Anakinra (%)	Intubated (%)	Male Sex (%)	Age (mean)	Symptoms Duration before Anakinra Administration (days; mean)	Baseline CRP (mg/L)	Oxygen Requirements (%)	HR for Death (95% CI) (Treated with Anakinra vs. Not)
Kyriazopoulou et al. [25]	Double-blind RCT	594	68	0	58	62	9	51	No oxygen: 8% Low-flow oxygen: 92%	0.45 (0.21, 0.98)
Kyriazopoulou et al. [23]	NR	260	50	0	63	64	7	47	No oxygen: 45% Oxygen: 55%	0.49 (0.25, 0.97)
Cavalli et al. [22]	NR	337	18	0	75	67	11	143	Oxygen 82% NIMV: 18%	0.45 (0.20, 0.99)
Pontali et al. (early) [24]	NR	107	59	7	69	63	9	87	Not reported: 67% NIMV: 24% IMV: 9%	0.33 (0.10, 1.12)
Pontali et al. (late) [24]	NR	65	32	8	69	68	15	67		0.82 (0.30, 2.27)
CORIMUNO-19 Collaborative group [21]	R	114	52	0	70	67	10	121	Low-flow Oxygen: 100%	0.77 (0.33, 1.77)
Bozzi et al. [20]	NR	120	54	33	80	62	12	148	Oxygen: 67% IMV: 33%	0.18 (0.07, 0.50)

CI: confidence intervals; CRP: c-reactive protein; HR: hazard ratio; IMV: invasive mechanical ventilation; NIMV: non-invasive mechanical ventilation; NR: non-randomized; R: randomized non-controlled; RCT: randomized-controlled trial.

## Data Availability

The data that support the findings of this study are available from the corresponding author (K.G.K.) upon reasonable request.

## Supplementary Materials

The following are available online at https://www.mdpi.com/article/10.3390/jcm10194462/s1, Figure S1: The PRISMA 2020 flow diagram for updated systematic reviews and meta-analyses study selection, Figure S2: The assessment of the risk of bias of the included studies, Table S1: The PRISMA 2020 Checklist for the present meta-analysis, Table S2: The PRISMA 2020 for Abstracts Checklist for the present meta-analysis, Table S3: The assessment of the risk of bias of the included studies for the present meta-analysis using a checklist from Joanna Briggs Institute Critical Appraisal Checklists for Cohort Studies, Table S4: Certainty of the evidence on the outcome of death for the present meta-analysis using the GRADE approach.
